# Novel models by machine learning to predict prognosis of breast cancer brain metastases

**DOI:** 10.1186/s12967-023-04277-2

**Published:** 2023-06-21

**Authors:** Chaofan Li, Mengjie Liu, Yinbin Zhang, Yusheng Wang, Jia Li, Shiyu Sun, Xuanyu Liu, Huizi Wu, Cong Feng, Peizhuo Yao, Yiwei Jia, Yu Zhang, Xinyu Wei, Fei Wu, Chong Du, Xixi Zhao, Shuqun Zhang, Jingkun Qu

**Affiliations:** 1 Department of Oncology, The Second Affiliated Hospital of Xi’an Jiaotong University, 157 West Fifth Street, Xi’an, Shaanxi People’s Republic of China; 2 Department of Otolaryngology, The Second Affiliated Hospital of Xi’an Jiaotong University, 157 West Fifth Street, Xi’an, Shaanxi People’s Republic of China; 3 Department of Radiation Oncology, The Second Affiliated Hospital of Xi’an Jiaotong University, 157 West Fifth Street, Xi’an, Shaanxi People’s Republic of China

**Keywords:** Breast cancer, Brain metastases, XGBoost algorithm, SEER, Surgery

## Abstract

**Background:**

Breast cancer brain metastases (BCBM) are the most fatal, with limited survival in all breast cancer distant metastases. These patients are deemed to be incurable. Thus, survival time is their foremost concern. However, there is a lack of accurate prediction models in the clinic. What’s more, primary surgery for BCBM patients is still controversial.

**Methods:**

The data used for analysis in this study was obtained from the SEER database (2010–2019). We made a COX regression analysis to identify prognostic factors of BCBM patients. Through cross-validation, we constructed XGBoost models to predict survival in patients with BCBM. Meanwhile, a BCBM cohort from our hospital was used to validate our models. We also investigated the prognosis of patients treated with surgery or not, using propensity score matching and K–M survival analysis. Our results were further validated by subgroup COX analysis in patients with different molecular subtypes.

**Results:**

The XGBoost models we created had high precision and correctness, and they were the most accurate models to predict the survival of BCBM patients (6-month AUC = 0.824, 1-year AUC = 0.813, 2-year AUC = 0.800 and 3-year survival AUC = 0.803). Moreover, the models still exhibited good performance in an externally independent dataset (6-month: AUC = 0.820; 1-year: AUC = 0.732; 2-year: AUC = 0.795; 3-year: AUC = 0.936). Then we used Shiny-Web tool to make our models be easily used from website. Interestingly, we found that the BCBM patients with an annual income of over USD$70,000 had better BCSS (HR = 0.523, 95%CI 0.273–0.999, P < 0.05) than those with less than USD$40,000. The results showed that in all distant metastasis sites, only lung metastasis was an independent poor prognostic factor for patients with BCBM (OS: HR = 1.606, 95%CI 1.157–2.230, P < 0.01; BCSS: HR = 1.698, 95%CI 1.219–2.365, P < 0.01), while bone, liver, distant lymph nodes and other metastases were not. We also found that surgical treatment significantly improved both OS and BCSS in BCBM patients with the HER2 + molecular subtypes and was beneficial to OS of the HR−/HER2− subtype. In contrast, surgery could not help BCBM patients with HR + /HER2− subtype improve their prognosis (OS: HR = 0.887, 95%CI 0.608–1.293, P = 0.510; BCSS: HR = 0.909, 95%CI 0.604–1.368, P = 0.630).

**Conclusion:**

We analyzed the clinical features of BCBM patients and constructed 4 machine-learning prognostic models to predict their survival. Our validation results indicate that these models should be highly reproducible in patients with BCBM. We also identified potential prognostic factors for BCBM patients and suggested that primary surgery might improve the survival of BCBM patients with HER2 + and triple-negative subtypes.

**Supplementary Information:**

The online version contains supplementary material available at 10.1186/s12967-023-04277-2.

## Introduction

Breast cancer (BC) is emerging as the top diagnosed cancer worldwide and the leading cause of cancer-related deaths in women [1]. BC metastasis to the central nervous system (CNS) is a devastating disease involving either the brain parenchyma or the leptomeninges. Of newly diagnosed BC patients annually, 10–16% will experience symptomatic brain metastases, and more than 30% of patients with metastatic BC are found in autopsy reports [2–5].

Patients with breast cancer brain metastases (BCBM) suffer from a particularly poor prognosis, with their median survival time being only 10 months [6]. Moreover, brain metastases usually lead to progressive neurologic deficits, which further reduce the quality of life [7]. Sadly, patients with BCBM are refractory to almost all currently available treatments, experiencing a traumatic deterioration of quality of life and a devastating < 20% 1-year survival [8]. A major reason for such a dreadful prognosis is that current treatment options for brain metastasis (e.g. steroids, cranial radiotherapy, and surgical resection in selected patients) are limited and merely palliative, not curative. Additionally, diverse clinical characteristics greatly affect the prognosis of BCBM patients [9]. Therefore, there is an urgent need for prognostic prediction models to accurately answer BCBM patients' concerns about survival and to help optimize their management.

Previous studies have built a few nomograms for predicting the prognosis of BCBM patients. To predict the prognosis of BCBM patients, a few nomograms have been developed in earlier investigations. These models' accuracy, however, is unsatisfactory (AUC value or C-index less than 0.7) [10–12]. Therefore, a more precise and robust model is required. To this end, machine learning has emerged as an absolutely crucial topic, offering tools and methods for evaluating the tremendous, high-dimensional, and multi-modal data generated by the biological sciences [13, 14]. It can also help us create an artificial intelligence (AI) prognostic model, significantly increasing the accuracy rate [14]. Extreme Gradient Boosting (XGBoost), one of the numerous machine learning algorithms, is created iteratively to minimize the loss function, which makes it perform well in various domains [15–17]. However, it is rarely applied in the prognostic prediction of cancer patients. We used 6 kinds of machine learning algorithms to create prognostic models and found that XGBoost performed best.

The Surveillance Epidemiology and End Results (SEER) database was exploited in this study to examine the variables affecting BCBM patients' prognoses. High-precision AI models were developed to predict the 6-month, 1, 2 and 3-year survival of BCBM patients. This study contributes to the development of clinical AI models to optimize the long-term follow-up of BCBM patients and provides insight into the prognosis of BCBM patients.

## Materials and methods

### Data source and study design

**Fig. 1:**
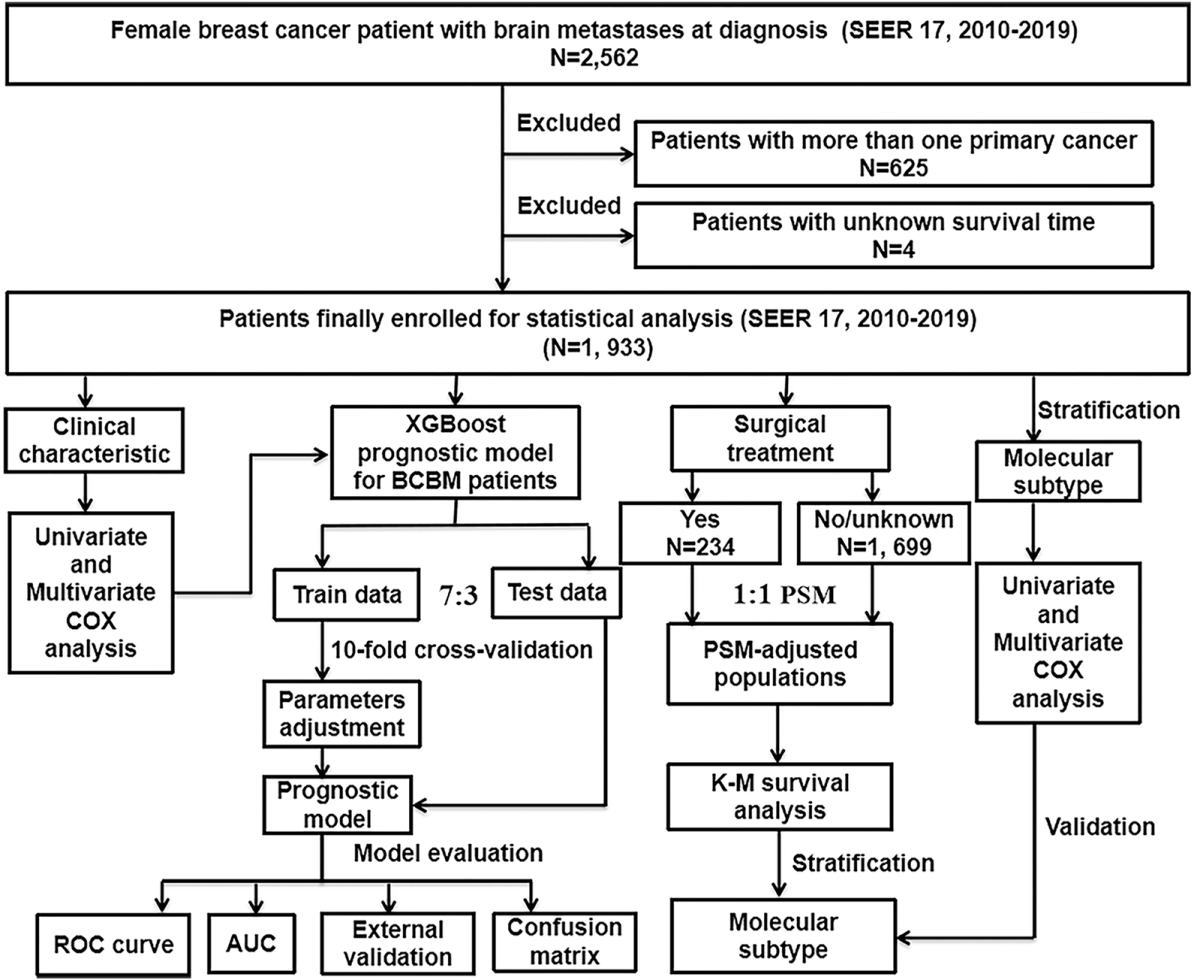
The flowchart described the process of conducting the study and statistical analysis. *SEER* the surveillance, epidemiology, and end results database; *BCBM* breast cancer brain metastases, *PSM* propensity score matching, *COX* concordance index; *ROC curve* receiver operating characteristic curve, *AUC* area under the curve, K–M Kaplan–Meier, *XGBoost* extreme gradient boosting

Figure 1 presents the workflow of our study design and its analyses. As the information on distant metastases was included from 2010, the data analyzed in this study were obtained from the SEER database [SEER 17 Regs study data, (changes 2010–2019); version 8.4.0] where the data is openly accessible. Data about women with BC were collected from this database. Inclusion criteria were as follows: (1) BC was the patients’ one and only cancer that had been identified; (2) all cancer patients showed histopathological and morphological evidence in accordance with the International Classification of Cancer Diseases Edition III (ICD-O-3); (3) all cancer patients developed brain metastases at the initial diagnosis. Exclusion criteria were as follows: (1) patients suffering from two or more primary cancers; (2) patients whose survival time was unknown. Follow up is sustained until patients died, loss to follow-up, or December 31, 2019.

### XGBoost model

The XGBoost algorithm modifies the gradient boosting approach by utilizing Newton's method to solve for the extreme values of the loss function, conducting Taylor expansion of the loss function to the second order, and adding a regularization term to the loss function The gradient boosting algorithm loss and the regularization term make up the first and second parts of the objective function at training time, respectively. In addition, the XGBoost algorithm adopts a technique named "feature subsampling”, which can be understood as selecting a subset of all features to train each tree (similar to a random forest) so as to improve the generalization ability of the model, make it more diverse and prevent overfitting. The XGBoost algorithm operates under the following principle: feature vector with the corresponding (output) category yi:

$${\text{yi = }}\sum\limits_{{\text{k = 1}}} {{\text{Kfk(xi)}}} {\text{,}}\,{{\text{f}}_{\text{k}}} \in {\text{F,}}$$

Feature selection: univariate and multivariate COX analyses were performed on clinical characteristics obtained from the SEER database. Characteristics that were statistically significant in the multivariate COX, including age at diagnosis, marital status, histological type, molecular subtype, T stage, lung metastases and chemotherapy, median household income,, as well as grade, race, surgery, radiotherapy, liver metastases reported as independent prognostic factors in previous studies [10, 18–20], were incorporated into machine learning models to predict 6-month, 1-, 2- and 3-year overall BCBM patient survival. Prior to excluding the patients who survived but lived less than 6-month, 1-, 2- or 3-year at the follow-up cut-off date, these analyses were conducted. A response variable was collected for survival information before running the training program, in which 1 = survival and 0 = death. Patients were randomized into train data and test data in a 7:3 ratio. We also compared the area under the curve (AUC value) of logistic regression (LR), support vector machine (SVM), random forest (RF), K-Nearest Neighbor (KNN), decision tree (ID3), and XGBoost on test data. Receiver operating characteristic (ROC) analysis, area under the ROC curve (AUC) and confusion matrix were used to evaluate the model. Precision and accuracy are the primary assessment parameters in the confusion matrix.

External validation: to further validate the XGBoost prognostic model, we collected information on 67 patients diagnosed with BCBM from May 2015 to May 2022 in the Second Affiliated Hospital of Xi’an Jiaotong University. Exclusion criteria were as follows: (1) under the age of 20; (2) patients with second primary cancer of any kind; (3) male BC patients; (4) patients who were lost to follow-up. Follow proceeded until the patient's death or November 5th, 2022. Our retrospective cohort study was authorized by the Institutional Review Board of the Second Affiliated Hospital of Xi’an Jiaotong University, which consented to waive informed consent because the data used in this study have no personally identifiable information of patients.

Shiny app: we built a web-based application to make our new predictive models available online. The web-based application was built based on the R package “shiny”.

### Statistical analysis

To explore the connection between various clinical and pathological features and the survival of patients, we sued univariate COX regression models. To assess patient mortality risk and identify independent prognostic markers, further multifactorial COX analysis was conducted. Patients undergoing surgical therapy and those who did not were matched on a 1:1 propensity score matching (PSM) based on the variables in the XGBoost model to examine the effect of surgical treatment on the prognosis of patients with BCBM. On the PSM-adjusted population, a Kaplan–Meier (K–M) survival analysis stratified by molecular subtype was also carried out. Finally, we performed subgroup univariate and multifactorial COX analyses in BCBM patients according to molecular subtype. We further investigated the role of treatment in patients with different molecular subtypes of BCBM. For all statistical calculations, the R programming language was utilized (version 4.0.2). Statistical significance was defined as a bilateral tail value of less than 0.05.

## Results

### Clinical characteristics of BCBM patients

Eventually, we obtained the information on 1933 eligible BCBM patients from the SEER database (2010 to 2019). The clinicopathological characteristics of BC patients with brain metastases are shown in Table 1 and summarized below. The median age of the patients was 60 years, of which 141 (7.29%) patients were younger than 40 years, and 129 (6.67%) patients were older than 80 years. While 739 patients (38.23%) received therapy more than a month following diagnosis, 877 patients (45.37%) received immediate medical attention. HR + /HER2− made up 37.09% of the molecular subtypes,, followed by HR−/HER2− (17.23%), HR + /HER2 + (15.93%) and HR−/HER2 + (12.36%). In terms of race, 74.86% of the patients were white. Invasive ductal carcinoma (IDC) was the predominant histopathological type (65.13%). Regarding marital status, 40.87% of the patients were married, and 25.61% were single. The proportions of staging T1 to T4 were 10.14%, 21.31%, 12.83% and 33.11%, respectively and N0 to N3 were 19.56%, 41.49%, 8.85% and 14.49%. Approximately 39.11% of the patients with tumors progressed to grade III or IV tumors, while only 3.47% had grade I. About 34.97% of the patients had a good annual family income of US$70,000. In the treatment field, only 12.11% of patients received surgery, 60.84% received radiotherapy, and 54.58% received chemotherapy. Bone, liver, and lung metastases, distant lymph nodes and other distant organ metastases accounted for 64.83%, 33.32%, 43.51%, 14.69% and 11.02% of patients, respectively.

**Table 1:** Baseline characteristics of BC brain metastases (BCBM) patients included from SEER data cohort

Characteristic		Cases	%
Age at diagnosis	< 40	141	7.29
	40–49	251	12.98
	50–59	529	27.37
	60–69	576	29.80
	70–79	307	15.88
	80 +	129	6.67
Months from diagnosis to therapy	0 month	877	45.37
	≥ 1 month	739	38.23
	Unknown	317	16.40
Subtype	HR + /HER2−	717	37.09
	HR + /HER2 +	308	15.93
	HR−/HER2 +	239	12.36
	HR−/HER2−	333	17.23
	Unknown	336	17.38
Race	White	1447	74.86
	Black	307	15.88
	Other	179	9.26
Histological type	IDC	1259	65.13
	ILC	97	5.02
	Mixed	70	3.62
	Other	507	26.23
Marital status	Married	790	40.87
	Singled	495	25.61
	Widow/divorced/other	648	33.52
T Stage	T1	196	10.14
	T2	412	21.31
	T3	248	12.83
	T4	640	33.11
	Unknown	437	22.61
N Stage	N0	378	19.56
	N1	802	41.49
	N2	171	8.85
	N3	280	14.49
	Unknown	302	15.62
Grade	Well differentiated	67	3.47
	Moderate differentiated	448	23.18
	Poorly differentiated	756	39.11
	Unknown	662	34.25
Median household income(inflation adjusted)	< 40,000$	104	5.38
	40,000–49,999$	219	11.33
	50,000–59,999$	292	15.11
	60,000–69,999$	642	33.21
	70,000$ +	676	34.97
Chemotherapy	No/unknown	878	45.42
	Yes	1055	54.58
Radiotherapy	No/unknown	757	39.16
	Yes	1176	60.84
Surgery	No/unknown	1699	87.89
	Yes	234	12.11
Bone metastases	No/unknown	680	35.18
	Yes	1253	64.82
Liver metastases	No/unknown	1289	66.68
	Yes	644	33.32
Lung metastases	No/unknown	1092	56.49
	Yes	841	43.51
Distant lymph nodes metastases	No/unknown	1649	85.31
	Yes	284	14.69
Distant other metastases	No/unknown	1720	88.98
	Yes	213	11.02

### Univariable and multivariable COX regression analysis

We practiced univariable COX regression analysis to spot variables that significantly influenced overall survival (OS) and breast cancer specific survival (BCSS) of BCBM patients, including age at diagnosis, race, marital status, histological type, months from diagnosis to therapy, median family income (inflation-adjusted), molecular subtype, T and N stage, grade, distant metastases and treatment information (Table 2).

**Table 2:** Univariate and multivariate COX analysis of characteristics extracted from SEER database

	Univariate COX analysis						Multivariate COX analysis					
	OS			BCSS			OS			BCSS		
	HR	95%CI	P Value	HR	95%CI	P Value	HR	95%CI	P Value	HR	95%CI	P Value
Age at diagnosis												
< 40	Reference			Reference			Reference			Reference		
40–49	1.292	1.006–1.660	*	1.201	0.922–1.564	0.175	1.322	0.646–2.706	0.444	1.105	0.521–2.279	0.791
50–59	1.423	1.132–1.788	**	1.371	1.080–1.740	**	2.232	1.180–4.221	*	2.293	1.165–4.101	*
60–69	1.674	1.336–2.099	***	1.608	1.270–2.037	***	1.900	1.030–3.620	*	1.746	1.002–3.181	*
70–79	2.291	1.802–2.911	***	2.175	1.692–2.796	***	3.336	1.636–6.805	***	3.737	1.845–7.569	***
80 +	2.710	2.056–3.574	***	2.448	1.824–3.286	***	1.908	0.764–4.767	0.166	2.282	1.061–5.617	*
Months from diagnosis to therapy												
0 month	Reference			Reference			Reference			Reference		
≥ 1 month	0.842	0.753–0.942	**	0.846	0.751–0.953	**	0.799	0.580–1.088	0.159	0.782	0.569–1.075	0.128
Subtype												
HR + /HER2-	Reference			Reference			Reference			Reference		
HR + /HER2 +	0.772	0.659–0.904	**	0.790	0.668–0.934	**	0.900	0.559–1.497	0.674	0.860	0.516–1.435	0.564
HR-/HER2 +	0.962	0.813–1.138	0.653	0.983	0.822–1.174	0.846	2.163	1.407–3.606	***	2.029	1.251–3.290	**
HR-/HER2-	1.744	1.511–2.015	***	1.830	1.572–2.129	***	3.575	2.380–5.771	***	3.369	2.150–5.278	***
Race												
White	Reference			Reference			Reference			Reference		
Black	1.266	1.107–1.448	***	1.222	1.057–1.412	**	1.002	0.601–1.496	0.994	0.936	0.581–1.510	0.787
Other	0.883	0.735–1.061	0.185	0.812	0.663–0.993	*	0.824	0.480–1.541	0.519	0.754	0.411–1.382	0.361
Histological type												
IDC	Reference			Reference			Reference			Reference		
ILC	0.992	0.779–1.262	0.947	0.955	0.738–1.236	0.728	2.794	1.266–6.197	*	2.838	1.297–6.214	**
Mixed	0.887	0.679–1.158	0.449	0.857	0.644–1.139	0.287	1.040	0.536–2.027	0.908	0.934	0.468–1.862	0.846
Other	1.354	1.209–1.516	***	1.225	1.083–1.386	**	1.422	0.784–2.724	0.270	1.015	0.484–2.128	0.970
Marital status												
Married	Reference			Reference			Reference			Reference		
Singled	1.262	1.111–1.435	***	1.251	1.091–1.435	**	1.793	1.198–2.695	**	1.879	1.253–2.819	**
Widow/divorced/other	1.519	1.351–1.706	***	1.531	1.353–1.734	***	1.571	1.034–2.253	*	1.543	1.044–2.279	*
T Stage												
T1	Reference			Reference			Reference			Reference		
T2	1.064	0.878–1.291	0.526	1.146	0.931–1.412	0.199	1.839	0.986–3.360	0.051	1.915	0.998–3.650	0.056
T3	1.137	0.921–1.405	0.233	1.231	0.981–1.545	0.073	1.652	0.784–3.289	0.168	1.543	0.756–3.146	0.233
T4	1.248	1.042–1.495	*	1.337	1.099–1.626	**	1.918	1.025–3.419	*	1.910	1.023–3.566	*
N Stage												
N0	Reference			Reference			refereNce			Reference		
N1	0.934	0.815–1.071	0.327	0.951	0.822–1.100	0.502	0.979	0.646–1.584	0.925	/	/	/
N2	0.788	0.645–0.963	*	0.834	0.675–1.029	0.09	0.864	0.411–1.702	0.687	/	/	/
N3	0.921	0.777–1.092	0.342	0.962	0.804–1.152	0.676	1.171	0.718–1.907	0.524	/	/	/
Grade												
Well differentiated	Reference			Reference			Reference			Reference		
Moderately differentiated	1.175	0.871–1.586	0.291	1.266	0.914–1.754	0.156	0.906	0.423–1.939	0.799	0.820	0.370–1.820	0.626
Poorly differentiated	1.435	1.072–1.921	*	1.561	1.137–2.145	**	1.140	0.529–2.458	0.738	1.177	0.530–2.618	0.689
Median household income(inflation adjusted)												
< 40,000$	Reference			Reference			Reference			Reference		
40,000–49,999$	1.055	0.823–1.352	0.676	1.006	0.774–1.308	0.965	0.559	0.254–1.230	0.148	0.604	0.277–1.320	0.207
50,000–59,999$	1.053	0.888–1.249	0.477	1.058	0.823–1.361	0.659	0.728	0.357–1.488	0.385	0.733	0.356–1.506	0.398
60,000–69,999$	0.931	0.805–1.076	0.748	0.936	0.742–1.181	0.579	0.724	0.381–1.377	0.325	0.651	0.341–1.243	0.193
70,000$ +	0.760	0.656–0.879	*	0.751	0.595–0.948	*	0.522	0.271–1.006	0.052	0.523	0.273–0.999	*
Chemotherapy												
No/unknown	Reference			Reference			Reference			Reference		
Yes	0.454	0.410–0.502	***	0.477	0.428–0.531	***	0.386	0.267–0.558	***	0.436	0.301–0.637	***
Radiotherapy												
No/unknown	Reference			Reference			Reference			Reference		
Yes	0.629	0.568–0.697	***	0.667	0.598–0.744	***	0.721	0.499–1.042	0.082	0.753	0.520–1.092	0.135
Surgery												
No/unknown	Reference			Reference			Reference			Reference		
Yes	0.619	0.529–0.724	***	0.615	0.520–0.727	***	0.693	0.430–1.117	0.132	0.742	0.464–1.186	0.212
Bone metastases												
No/unknown	Reference			Reference			Reference			Reference		
Yes	0.956	0.859–1.063	0.407	1.002	0.894–1.123	0.973	/	/	/	/	/	/
Liver metastases												
No/unknown	Reference			Reference			Reference			Reference		
Yes	1.290	1.159–1.436	***	1.283	1.144–1.438	***	1.286	0.892–1.860	0.179	1.264	0.877–1.823	0.209
Lung metastases												
No/unknown	Reference			Reference			Reference			Reference		
Yes	1.434	1.294–1.589	***	1.426	1.278–1.592	***	1.606	1.157–2.230	**	1.698	1.219–2.365	**
Distant Lymph nodes metastases												
No/unknown	Reference			Reference			Reference			Reference		
Yes	1.128	0.942–1.352	0.190	1.093	0.900–1.327	0.368	/	/	/	/	/	/
Distant other metastases												
No/unknown	Reference			Reference			Reference			Reference		
Yes	1.331	1.101–1.609	**	1.322	1.079–1.620	**	1.195	0.829–1.723	0.339	1.216	0.842–1.757	0.296

^*^P < 0.05,

^**^P < 0.01,

^***^P < 0.001

Then, we performed multivariable COX regression analysis to eliminate confounding factors and uncover the independent factors that influence OS and BCSS (Table 2). It showed that in patients aged > 50, ILC, T4 stage, lung metastases were greatly related to worse OS and BCSS. Patients with HR−/HER2 + and HR−/HER2-subtypes demonstrated poorer OS and BCSS than HR + /HER2-patients, whereas there was no difference between HR + /HER2- and HR + /HER2 + . In terms of treatment, it showed that only chemotherapy was able to prolong OS and BCSS in multivariable COX regression analysis rather than radiotherapy and primary tumor surgery. The prognosis was also influenced by a few social factors, including marital status and financial stability of the family. Married status and yearly household income of over USD$70,000 were tightly linked to higher survival.

### Establishing and evaluating predictive models for estimating the prognosis of patients with BCBM

**Fig. 2:**
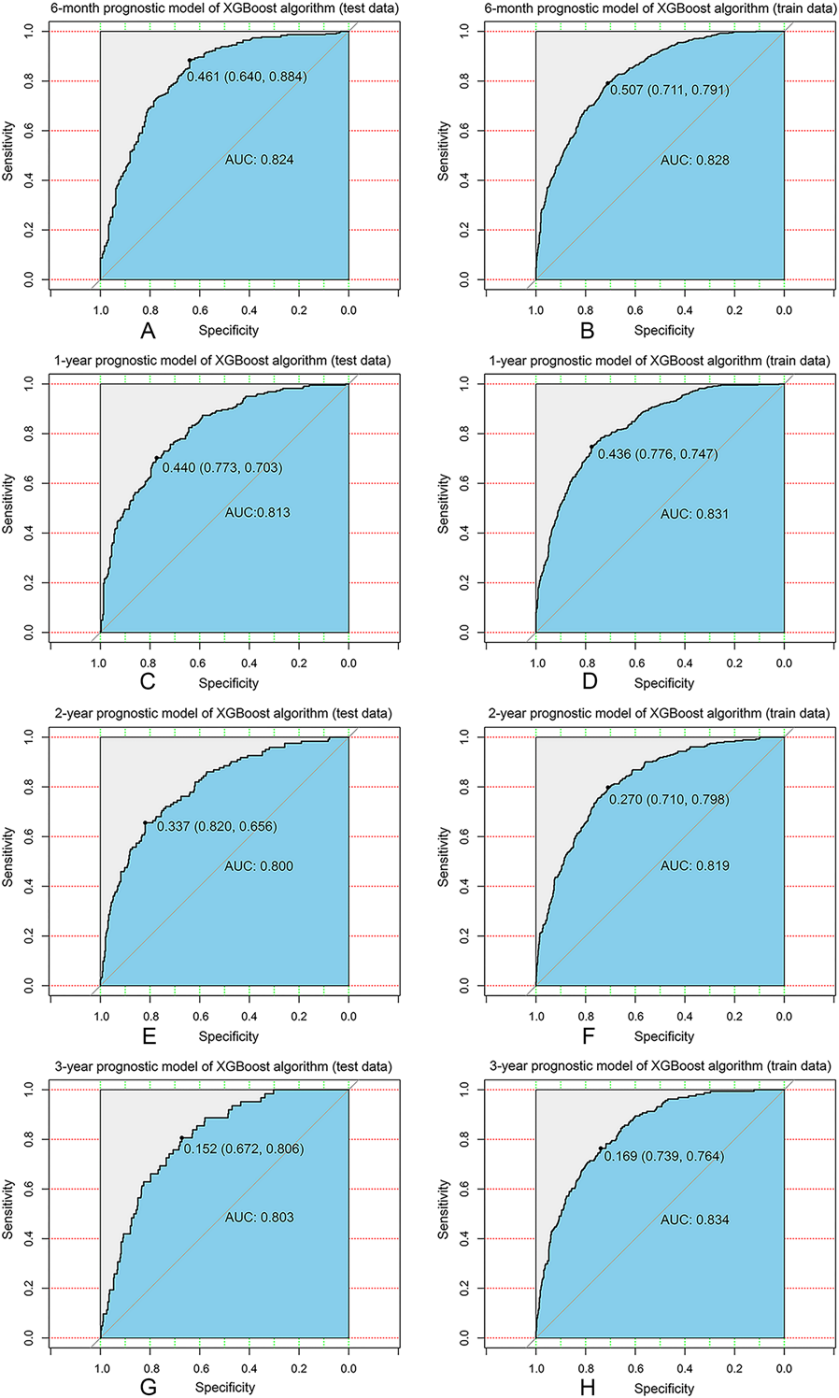
XGBoost model evaluation. **A** ROC curve for the 6-month prognostic model (test data); **B** ROC curve for the 6-month prognostic model (train data); **C** ROC curve for the 1-year prognostic model (test data); **D** ROC curve for the 1-year prognostic model (train data); **E** ROC curve for the 2-year prognostic model (test data); **F** ROC curve for the 2-year prognostic model (train data); **G** ROC curve for the 3-year prognostic model (test data); **H** ROC curve for the 3-year prognostic model (train data); *ROC* receiver operating characteristic curve, *AUC* area under the curve, *XGBoost* extreme Gradient Boosting

In light of the results obtained, we took steps to establish an XGBoost prediction model to predict the OS of BCBM patients at six months, one year, two years, and three years. We sorted the patients into train and test data group in a 7:3 ratio. And to ensure the stability of the model, we used ten-fold cross-validation in the training set for iterative testing and tuning so as to confirm the key hyperparameters and generate the optimal model (Table 3). For the train and validation sets, we formed the predicted ROC curves and computed the corresponding AUCs. Our XGBoost model performed exceptionally well in predicting survival of BCBM patients at 6-month (test set: AUC = 0.824; train set AUC = 0.828), 1-year (test set: AUC = 0.813; train set AUC = 0.831), 2-year (test set: AUC = 0.800; train set AUC = 0.819) and 3-year (test set: AUC = 0.803; train set AUC = 0.834) (Fig. 2). Compared to traditional machine learning algorithms, LR (6-month: AUC = 0.794; 1-year: AUC = 0.744; 2-year: AUC = 0.740; 3-year: AUC = 0.744), RF (6-month: AUC = 0.770; 1-year: AUC = 0.729; 2-year: AUC = 0.730; 3-year: AUC = 0.756), SVM (6-month: AUC = 0.730; 1-year: AUC = 0.647; 2-year: AUC = 0.525; 3-year: AUC = 0.509), KNN (6-month: AUC = 0.738; 1-year: AUC = 0.623; 2-year: AUC = 0.581; 3-year: AUC = 0.586) and ID3 (6-month: AUC = 0.692; 1-year: AUC = 0.628; 2-year: AUC = 0.685; 3-year: AUC = 0.639), XGBoost model performed best (Table 4).

**Table 3:** Main parameters of the XGBoost model

Parameter	Value
Gamma	1
Min_child_weight	10
Subsample	0.8
Max_delta_step	6
Alpha	2
Max_depth	5
Eta	0.17
nround	25

**Table 4:** Performance of prognostic models built by machine learning algorithms on test data (area under the ROC curve)

	6-month survival	1-year survival	2-year survival	3-year survival
XGBoost	0.824	0.813	0.800	0.803
LR	0.794	0.744	0.740	0.744
RF	0.770	0.729	0.730	0.756
SVM	0.730	0.647	0.525	0.509
KNN	0.738	0.623	0.581	0.586
ID3	0.692	0.628	0.685	0.639

*XGBoost* extreme gradient boosting, *LR* logistic regression, *RF* random forest, *SVM* support vector machine, *ID3* decision tree, *KNN* K-Nearest Neighbor

**Fig. 3:**
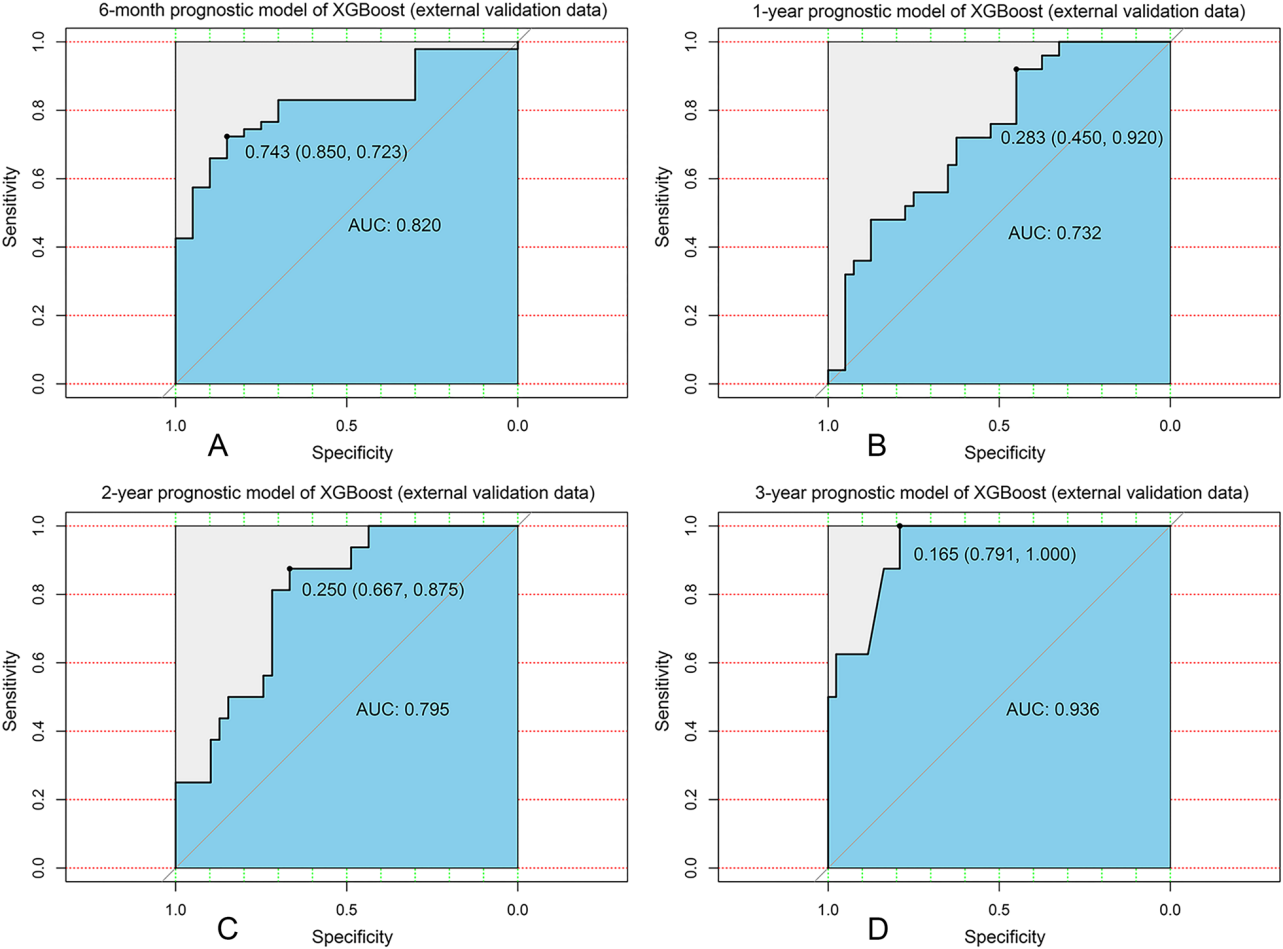
Validation of XGBoost models from external database. **A** ROC curve for the 6-month prognostic model (external validation data); **B** ROC curve for the 1-year prognostic model (external validation data); **C** ROC curve for the 2-year prognostic model (external validation data); **D** ROC curve for the 3-year prognostic model (external validation data); *ROC* receiver operating characteristic curve; *AUC* area under the curve; *XGBoost* extreme gradient boosting

In order to further validate our models, we collected clinical and prognostic information from 67 patients with BCBM from our hospital (Additional file 1: Table S1). It showed that our XGBoost models still exhibited good robustness in an externally independent dataset [6-month: AUC = 0.820 (Fig. 3A); 1-year: AUC = 0.732 (Fig. 3B); 2-year: AUC = 0.795 (Fig. 3C); 3-year: AUC = 0.936 (Fig. 3D)].

**Fig. 4:**
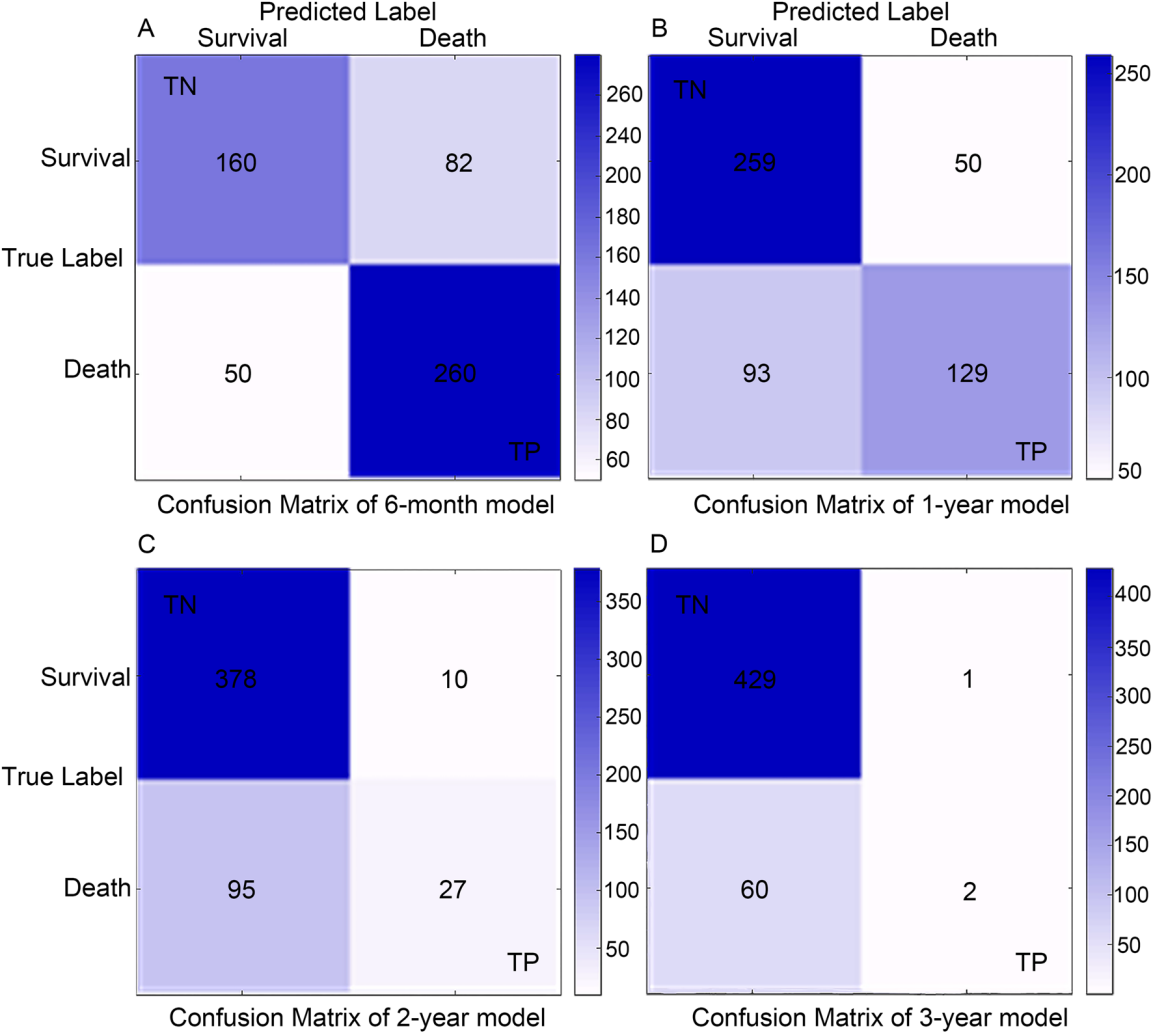
Confusion matrix of the XGBoost model’s predicted results in the test data. **A** Confusion matrix in the 6-month prognostic model; **B** confusion matrix in the 1-year prognostic model; **C** confusion matrix in the 2-year prognostic model; **D** confusion matrix in the 3-year prognostic model. *TP* true positive, *TN* true negative

Then, the effectiveness and precision of our XGBoost model was then assessed using a confusion matrix. The 6-month survival prediction model was calculated to have a correctness of 0.76 and a precision of 0.76 (Fig. 4A); the 1-year survival model had a correctness of 0.73 and a precision of 0.72 (Fig. 4B); the 2-year survival model had a correctness of 0.79 and a precision of 0.73 (Fig. 4C). And the 3-year survival model had a correctness of 0.88 and a precision of 0.67 (Fig. 4D). In general, our models behaved efficiently and successfully.

**Fig. 5:**
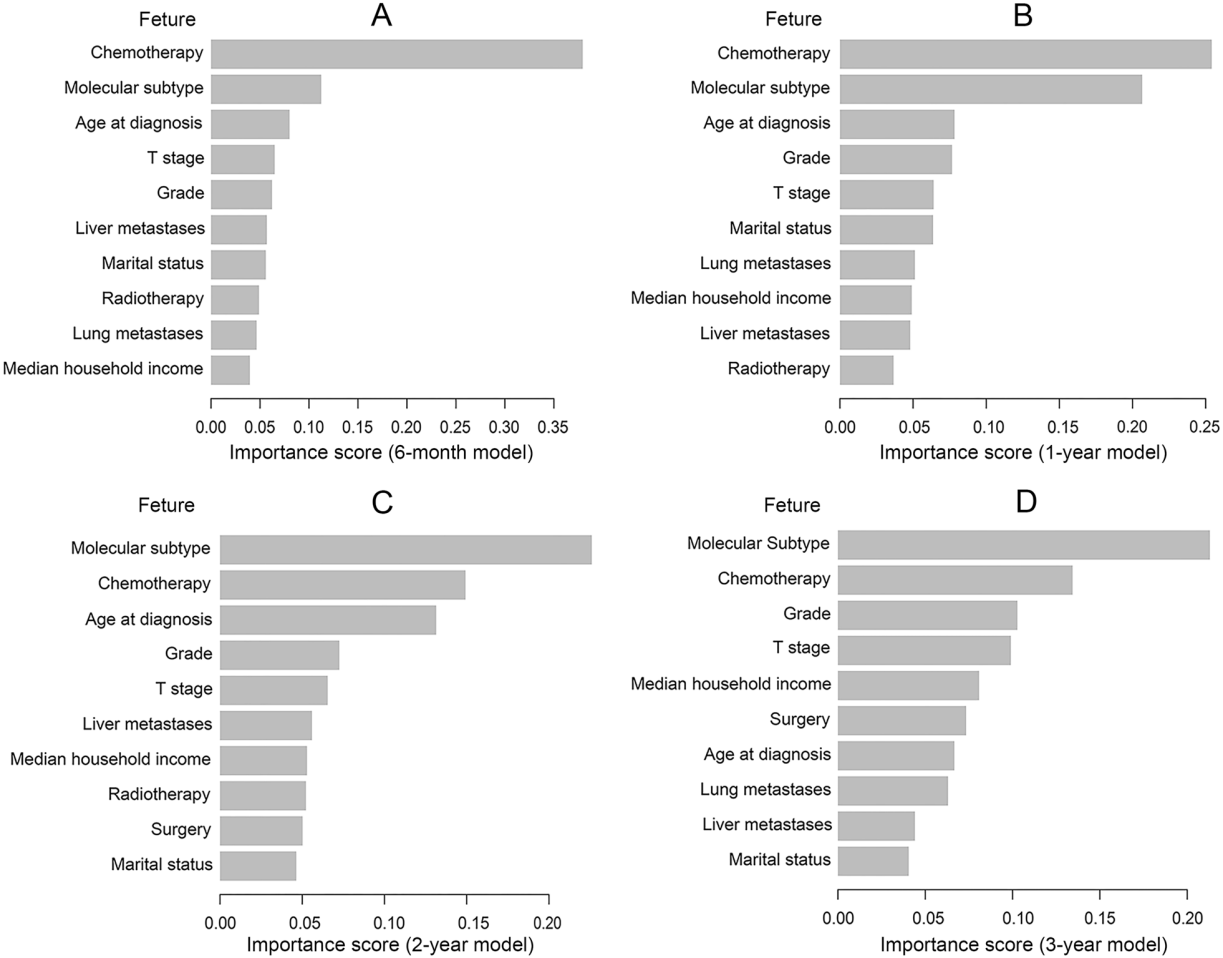
The ranking of clinical characteristics in terms of importance in the XGBoost prognostic model. **A** The ranking of clinical characteristics in terms of importance in the 6-month prognostic model; **B** the ranking of clinical characteristics in terms of importance in the 1-year prognostic model; **C** the ranking of clinical characteristics in terms of importance in the 2-year prognostic model; **D** the ranking of clinical characteristics in terms of importance in the 2-year prognostic model. XGBoost: extreme Gradient Boosting

Additionally, we graded how prominent clinical traits were in the models. The findings revealed that the top 5 factors affecting prognosis were chemotherapy, molecular subtype, age at diagnosis, grade and T stage. Among them, chemotherapy was the most important factor for short-term prognostic models (6-month and 1-year) (Fig. 5A and B), while molecular subtype was more important for medium- to long-term prognostic models (2 and 3-year) (Fig. 5C and D).

### Web-based application development

**Fig. 6:**
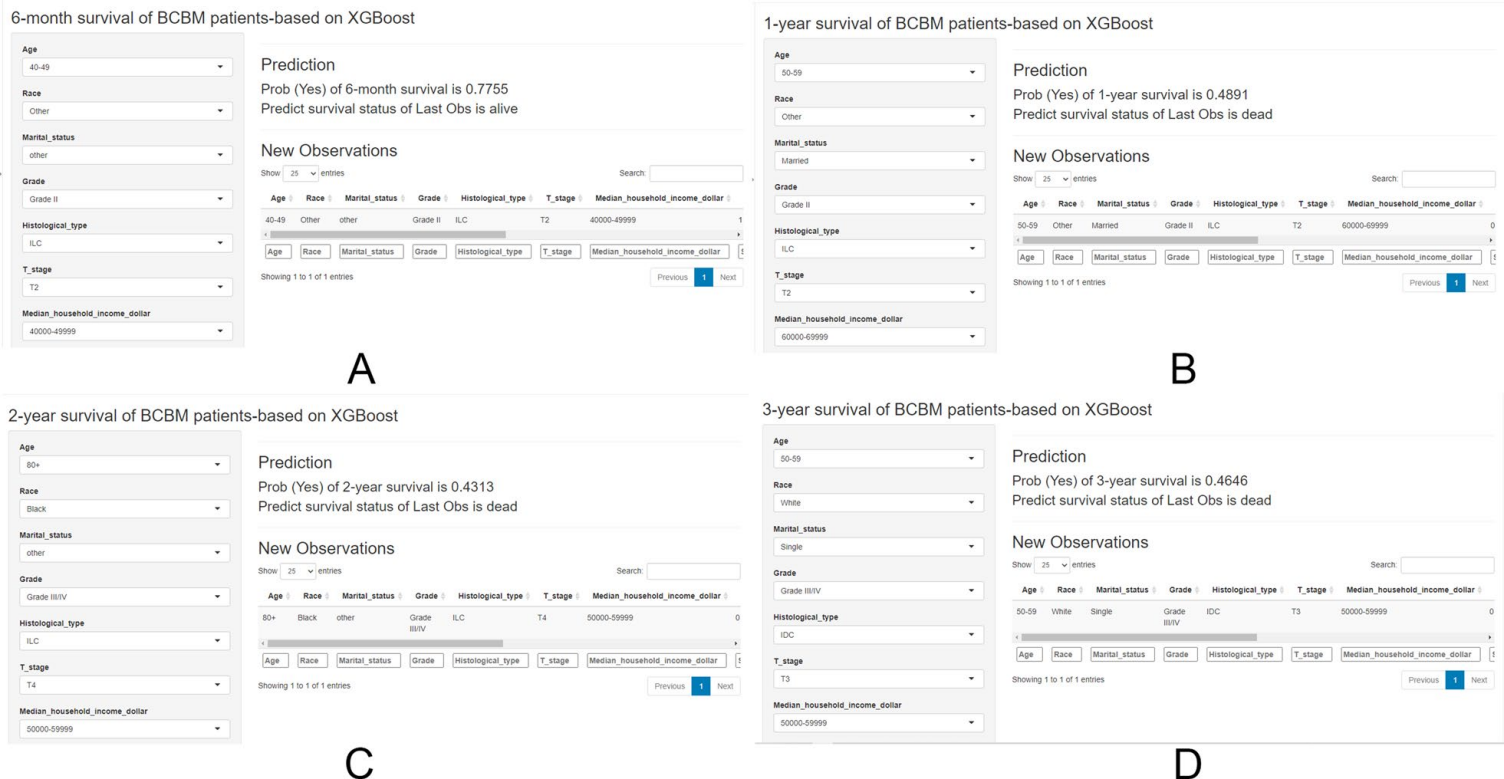
Screenshot of web app. **A** The screenshot of the 6-month prognostic model (https://lee2287171854.shinyapps.io/6-month_survival/); **B** the screenshot of the 1-year prognostic model (https://lee2287171854.shinyapps.io/1-year_survival/); **C** the screenshot of the 2-year prognostic model (https://lee2287171854.shinyapps.io/2-year_survival/); **D** the screenshot of the 3-year prognostic model (https://lee2287171854.shinyapps.io/3-year_survival/). XGBoost: extreme Gradient Boosting; BCBM: breast cancer brain metastases; NA: not applicable; 1 = yes, 0 = no; *HR ± * hormone receptor positive/negative, *HER2 ± * human epidermal growth factor receptor 2 positive/negative, *IDC* infiltrating ductal carcinoma, *ILC* infiltrating lobular carcinoma, Mixed: Infiltrating ductal and lobular carcinoma

To help researchers and clinicians learn to use our prognostic models, we have developed user-friendly web applications based on the shiny platform. The web interfaces (Fig. 6A–D) allow users to input clinical characteristics of a new sample and then the web application can help predict survival probabilities and survival status according to BCBM patient’s information.

### Benefits of surgical treatment in BCBM patients subdivided by molecular subtypes

Previous studies proved that surgical treatment was an independent prognostic factor for BCBM patients [10–12]. However, our multivariable COX regression analysis gave us the opposite result (Table2). Furthermore, we explored how surgery affected the prognosis of BCBM patients. Patients undergoing surgical therapy and those not undergoing surgery were compared based on their baseline characteristics (Table 5). These two groups had different baselines. Therefore, PSM was employed to adjust for the observed imbalance. After PSM correction, there were ultimately no significant differences in baseline characteristics (Table 5).

**Table 5:** Comparison of patient characteristics according to surgery treatment before and after propensity score matching (PSM)

Characteristics	Unmatched Cohort					1:1 propensity score matched (PSM) Cohort				
	Surgery		Surgery not given		Unadjusted	Surgery		Surgery not given		PSM-adjusted
	N = 234	%	N = 1699	%	P value	N = 200	%	N = 200	%	P value
Age at diagnosis					0.004					0.951
< 40	24	10.26	117	6.89		19	9.50	18	9.00	
40–49	41	17.52	210	12.36		34	17.00	32	16.00	
50–59	72	30.77	457	26.90		60	30.00	59	29.50	
60–69	57	24.36	519	30.55		49	24.50	50	25.00	
70–79	23	9.83	284	16.72		23	11.50	29	14.50	
80 +	17	7.26	112	6.59		15	7.50	12	6.00	
Subtype					< 0.001					0.815
HR + /HER2-	85	36.32	632	37.20		75	37.50	68	34.00	
HR + /HER2 +	33	14.10	275	16.19		30	15.00	29	14.50	
HR-/HER2 +	29	12.39	210	12.36		26	13.00	29	14.50	
HR-/HER2-	64	27.35	269	15.83		48	24.00	46	23.00	
Unknown	23	9.83	313	18.42		21	10.50	28	14.00	
Race					0.830					0.786
White	172	73.50	1275	75.04		143	71.50	148	74.00	
Black	38	16.24	269	15.83		34	17.00	33	16.50	
Other	24	10.26	155	9.12		23	11.50	19	9.50	
Histological type					< 0.001					0.700
IDC	177	75.64	1082	63.68		149	74.50	149	74.50	
ILC	11	4.70	86	5.06		10	5.00	6	3.00	
Mixed	12	5.13	58	3.41		10	5.00	9	4.50	
Other	34	14.53	473	27.84		31	15.50	36	18.00	
Marital status					0.132					0.623
Married	105	44.87	685	40.32		86	43.00	81	40.50	
Single	64	27.35	430	25.31		56	28.00	52	26.00	
Others	65	27.78	584	34.37		58	29.00	67	33.50	
T stage					< 0.001					0.802
T1	34	14.53	162	9.54		25	12.50	28	14.00	
T2	69	29.49	343	20.19		53	26.50	48	24.00	
T3	31	13.25	217	12.77		29	14.50	23	11.50	
T4	84	35.90	556	32.73		77	38.50	86	43.00	
Tx	16	6.84	421	24.78		16	8.00	15	7.50	
Grade					< 0.001					0.923
Well	9	3.85	58	3.41		8	4.00	7	3.50	
Moderately	52	22.22	396	23.31		48	24.00	43	21.50	
Poorly	146	62.39	610	35.90		117	58.50	121	60.50	
Unknown	27	11.54	635	37.37		27	13.50	29	14.50	
Median household income (inflation adjusted)					0.093					0.944
< 40,000$	19	8.12	85	5.00		15	7.50	14	7.00	
40,000–49,999$	30	12.82	189	11.12		24	12.00	28	14.00	
50,000–59,999$	41	17.52	251	14.77		35	17.50	30	15.00	
60,000–69,999$	76	32.48	566	33.31		63	31.50	63	31.50	
70,000$ +	68	29.06	608	35.79		63	31.50	65	32.50	
Chemotherapy					< 0.001					0.586
No/unknown	66	28.21	812	47.79		63	31.50	58	29.00	
Yes	168	71.79	887	52.21		137	68.50	142	71.00	
Radiotherapy					< 0.001					0.517
No/unknown	60	25.64	697	41.02		59	29.50	65	32.50	
Yes	174	74.36	1002	58.98		141	70.50	135	67.50	
Liver metastases					< 0.001					0.370
No/unknown	193	82.48	1096	64.51		159	79.50	166	83.00	
Yes	41	17.52	603	35.49		41	20.50	34	17.00	
Lung metastases					0.002					0.918
No/unknown	154	65.81	938	55.21		124	62.00	123	61.50	
Yes	80	34.19	761	44.79		76	38.00	77	38.50	

**Fig. 7:**
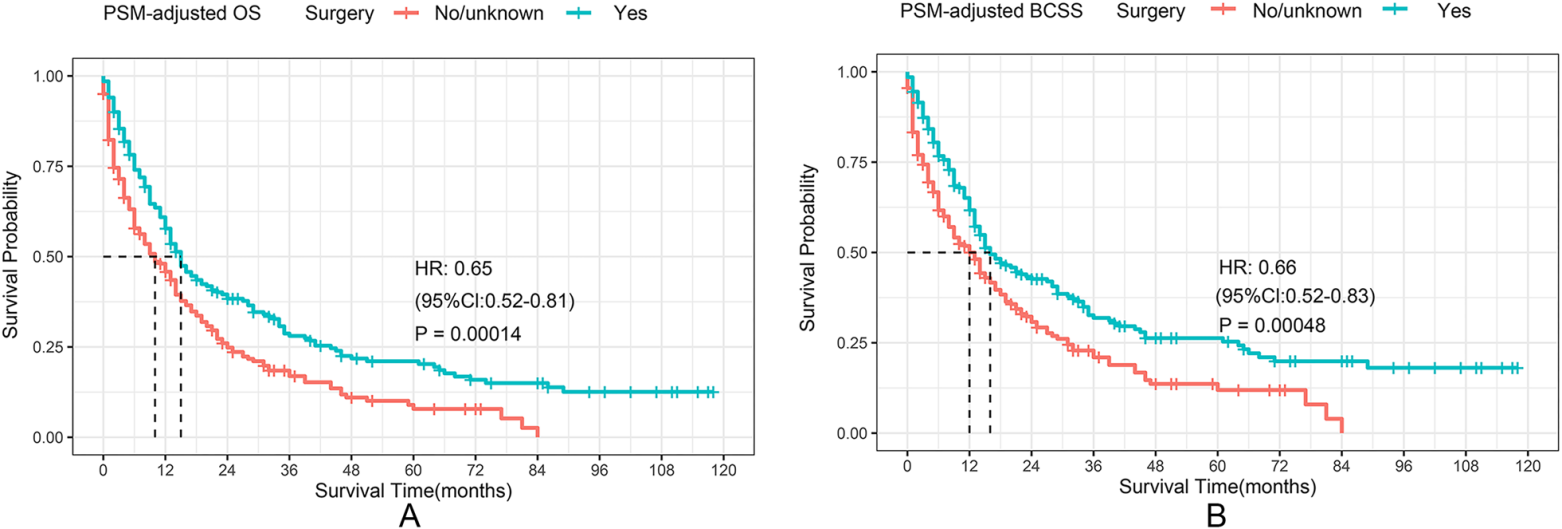
PSM adjusted OS and BCSS of BCBM patients with surgical treatment. Kaplan–Meier (K–M) survival analysis: **A** unadjusted OS of BCBM patients with surgical treatment; **B** PSM adjusted OS of BCBM patients with surgical treatment. *PSM* propensity score matching, *OS* overall survival; *BCBM* BC brain metastases, *HR* hazard ratio, *CI* confidence interval

**Fig. 8:**
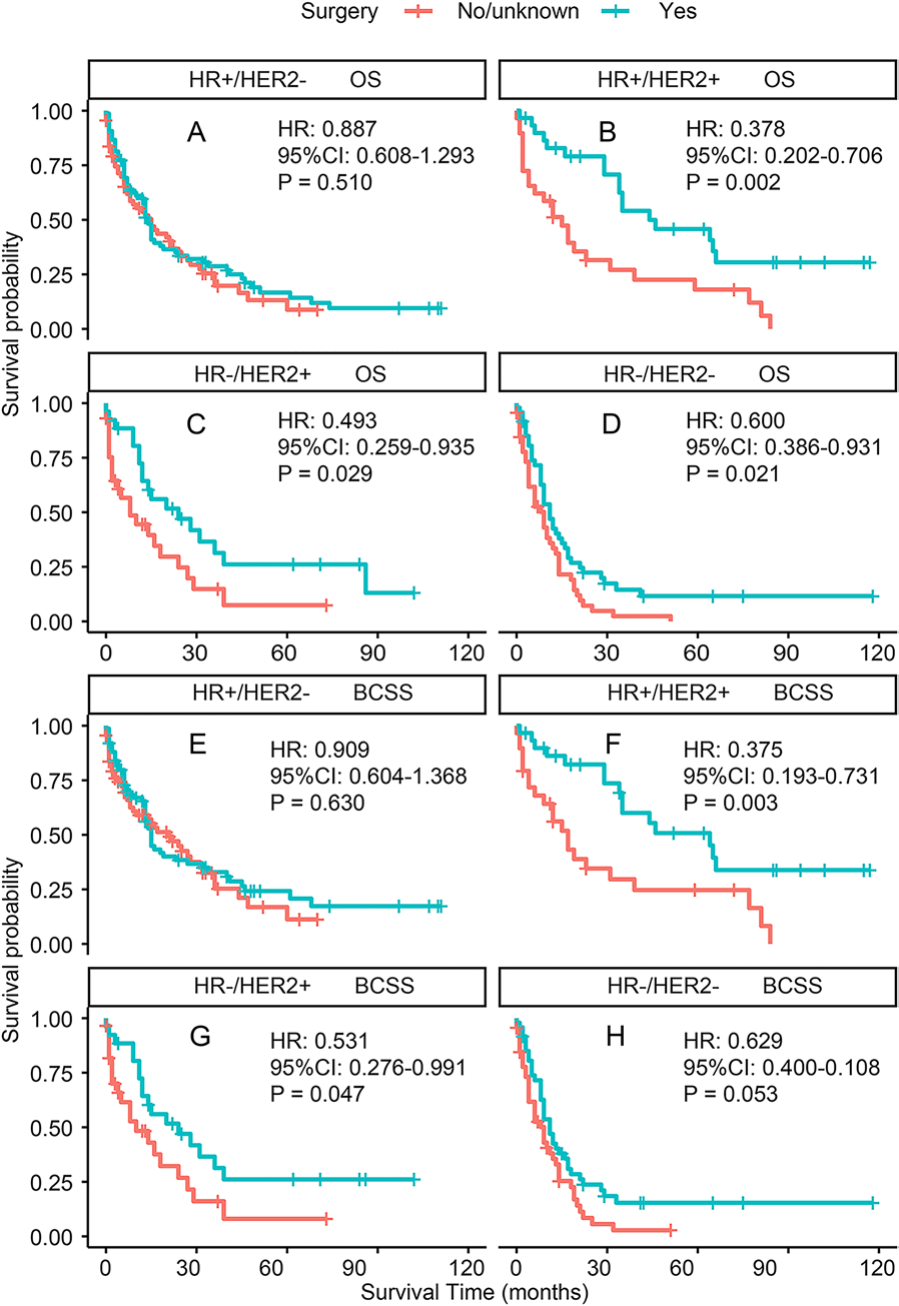
PSM adjusted OS and BCSS of BCBM patients with surgical treatment (Stratified by molecular subtype). Kaplan–Meier (K–M) survival analysis: **A** OS of BCBM patients with HR + /HER2− subtype; **B** OS of BCBM patients with HR + /HER2 + subtype; **C** OS of BCBM patients with HR−/HER2 + subtype; **D** OS of BCBM patients with HR−/HER2− subtype; **E** BCSS of BCBM patients with HR + /HER2− subtype; **F** BCSS of BCBM patients with HR + /HER2 + subtype; **G** BCSS of BCBM patients with HR−/HER2 + subtype; **H** BCSS of BCBM patients with HR−/HER2− subtype. *OS* overall survival, *BCSS* BC-specific survival, *BCBM* BC brain metastases, *HR ± * hormone receptor positive/negative, *HER2 ± * human epidermal growth factor receptor 2 positive/negative, *PSM* propensity score matching, *HR* hazard ratio, *CI* confidence interval

A 35% decrease in the overall risk of mortality in the surgery was observed in the PSM-adjusted data group (P = 0.00014, HR: 0.65; 95% CI 0.52–0.81) (Fig. 7A), with a similar reduction in the risk of BC-related death of approximately 34% (P = 0.00048, HR: 0.66; 95% CI 0.52–0.83) (Fig. 7B). The OS and BCSS of the BC patients with HR + /HER2 + and HR−/HER2 + subtypes enormously improved after surgery, according to the stratified K–M survival analysis. (Fig. 8B, C, F, G). However, no significant difference in HR + /HER2− subtype can be found (Fig. 8A, E). In addition, the effect of surgical treatment on OS and BCSS in patients with HR−/HER2− subtypes was different. To further validate these results, we divided all the 1933 eligible BCBM patients into four groups according to molecular subtype and performed univariate and multivariable COX analyses again (Additional file 2: Table S2). It showed that only HR + /HER2− subtype could not benefit from surgical treatment, which was consistent with our results of the PSM-adjusted K–M survival analysis.

## Discussion

The bone, lung, brain and liver etc. are the organs where BC might metastasis with a high probability of success. Different patient prognoses and reactions to therapy result from this organotropism [21]. Brain metastases are the most fatal. For these BCBM patients deemed, incurable, survival time is their foremost concern. The clinic practice, however, lacks reliable predictive models. In recent investigations, multiple nomogram prediction models for BCBM patients were constructed with the help of SEER datasets, but their accuracy rates are all less than 70% [10–12]. In consequence, more accurate and powerful models are needed. To our knowledge, the current study is the largest one to analyze the clinical characteristics and prognosis of BCBM patients. The 6-month, 1-, 2-, and 3-year OS of BCBM patients is 54.44% %, 40.51%, 23.78% and 13.61%, respectively. This study is the first one to create AI prognostic models for BCBM patients, and the models we made are the most accurate in predicting the survival of BCBM patients. In practice, our XGBoost models still exhibited good performance in an externally independent dataset. This demonstrates the high clinical utility of the models. Moreover, we have also created the first model for predicting the 3-year survival of BCBM patients with high accuracy.

This study identified several independent factors associated with better prognosis, including age < 50, HR + molecular subtype, IDC, married, low T stage, median household income over USD$70,000 and chemotherapy. Age > 40 years was a risk factor for BCBM patients to experience a worse OS, according to previous research [10, 18], whereas age 45–64 years was also a risk factor [12]. We analyzed more age groups and found that age > 50 was a feature for worse OS and BCSS. Compared to the HR + subtype, the patients with the HR− subtype showed poorer survival, similar to several previous studies [10, 11], and implied the importance of endocrine therapy for HR + BCBM patients. According to the research, the survival of BC patients could be impacted by household income [22]. Generally, patients with higher incomes have better prognoses. The OS and BCSS of BCBM patients with incomes over USD $70,000 were shown to be superior to those with incomes under USD $40,000 in our study. No income level boundary among BC patients was documented previously, while this may be a reflection of how well they cooperate with doctors throughout treatment. Several studies showed that extracranial organ metastases worsened the prognosis of patients with BCBM [10, 23]. Our study found that only lung metastasis is an independent poor prognostic factor for patients with BCBM, while bone, liver, distant lymph nodes and other metastases were not. In contrast, two previous studies indicated that liver metastasis was also an independent factor of BCBM patients [19, 20], but their studies only covered about 700 patients, which was much smaller than ours and incorporated fewer factors. For example, the study by Leone et al. did not even include chemotherapy as an important factor [20].

In terms of treatment, our analysis showed that only chemotherapy was an independent protective factor for all BCBM patients. Consistent with previous studies[10–12, 19], we also found radiotherapy was not an independent prognostic factor for BCBM patients, which further validated the effect of chemotherapy and radiotherapy on OS and BCSS of BCBM. One controversial topic is whether surgical therapy for the primary site improves the survival of BCBM patients. Previous studies showed that surgical treatment was an independent prognostic factor for BCBM patients [10–12]. However, our result was exactly the opposite of it, and another study indicated that surgical therapy, with the exception of brain metastases, positively affected the prognosis in primary metastatic BC patients with a single distant metastasis. [24]. Whether surgical therapy for the primary site prolongs survival time in patients with de novo metastatic BC has long been debatable, but current results imply that in well-selected patients, primary surgery might be a therapeutic option [25–33]. To more explicitly categorize the patients, we subsequently looked into the impact of surgery on the prognosis of BCBM patients with various molecular subtypes. In BCBM patients with HER2 + molecular subtypes, it was found that surgical intervention dramatically enhanced both OS and BCSS, suggesting that anti-HER2-targeted therapy combined with surgical treatment may prolong the survival of BCBM patients. We also found that for BCBM patients with HR-/HER2- subtype, the OS, but not BCSS, could benefit from surgery. In contrast, surgery could not help BCBM patients with HR + /HER2− subtype improve their prognosis, suggesting that chemotherapy and endocrine therapy are more important for these patients. Our findings suggested the necessity of surgery for HER2 + and triple-negative BCs (TNBC), which had the greatest incidence of brain metastases, compared with other BC subtypes [34, 35].

Our study may have some potential limitations despite its promising discoveries. First, although the SEER database includes about 30% of the USA population, this study’s sample size was constrained because the SEER database only incorporates the clinical data on tumor subtypes and distant metastatic sites following 2010. Second, the SEER database can greatly represent the general situation, but due to ethnic differences, it may not always apply to Asian and especially the Chinese. Third, the SEER database does not incorporate data on disease recurrence or subsequent sites of metastases. Therefore, we could not go further and look into the patients who developed brain metastases later in their remaining years, which may potentially result in some bias. Fourth, elaborate information on treatments of patients with brain metastases is not collected in the SEER database. We were unable to go deeper on this consequently. Furthermore, despite the extraordinary accuracy the machine learning prognostic model has achieved, external validation could be strengthened so that the study results can be more reliable.

## Conclusion

In conclusion, we analyzed the clinical features of BCBM patients and constructed 4 machine-learning prognostic models to predict their survival. According to the findings of our validation, these models are considered to be highly reproducible in BCBM patients. We further revealed potential prognostic variables for BCBM patients, and the survival of BCBM patients with the HER2 + and triple-negative subtypes may be greatly improved by primary surgery.

## Supplementary Information

**Additional file 1: Table S1.** Clinical and prognostic information from patients with BCBM from our hospital.  

**Additional file 2:** **Table S2.** Univariate and multivariate COX analysis of characteristics (stratified by molecular subtype).

## Data Availability

All data here are publicly available in the SEER database [https://seer.cancer.gov/ (accessed on April 15, 2022)].

## Abbreviations

BCBM: Breast cancer brain metastases
SEER: Surveillance, epidemiology, and end results
AUC: Area under the curve
COX: Concordance index
PSM: Propensity score matching
HR: Hazard ratio
CI: Confidence interval
OS: Overall survival
BCSS: Breast cancer-specific survival
HR ±: Hormone receptor positive/negative
HER2: Human epidermal growth factor receptor 2
BC: Breast cancer
LR: Logistic regression
SVM: Support vector machine
RF: Random forest
KNN: K-nearest neighbor
ID3: Decision tree
AI: Artificial intelligence
ICD-O-3: International classification of cancer diseases edition III
